# Lupus-Associated Knee Pain: An Atypical Presentation of Systemic Lupus Erythematosus in a Young Male

**DOI:** 10.7759/cureus.99557

**Published:** 2025-12-18

**Authors:** António L Pessoa, Ana Teixeira Reis, Ana Santos Costa, Miguel Rodrigues, Carolina De Almeida Robalo

**Affiliations:** 1 Internal Medicine, Centro Hospitalar de Setúbal, E.P.E., Setúbal, PRT

**Keywords:** alopecia areata (aa), auto-immune disease, patellofemoral chondromalacia, pet-scanner, systematic lupus erythematoses

## Abstract

Systemic lupus erythematosus (SLE) predominantly affects young women, and its presentation in male patients may be diagnostically challenging. We report a case of a 19-year-old male presenting with bilateral inflammatory knee pain initially interpreted as patellofemoral chondromalacia. ¹⁸F-fluorodeoxyglucose positron emission tomography-computed tomography (¹⁸F-FDG PET-CT) revealed multiple symmetric hypermetabolic osteo-medullary and subcutaneous lesions suggestive of systemic inflammation. Further evaluation demonstrated non-scarring alopecia, subcutaneous plaques, leukopenia, hypocomplementemia, and positive antinuclear antibodies (ANA), meeting the 2019 European Alliance of Associations for Rheumatology (EULAR)/American College of Rheumatology (ACR) criteria for SLE. Hydroxychloroquine initiation led to improvement in musculoskeletal symptoms. This case illustrates the importance of considering SLE in atypical demographics and in patients presenting with unexplained inflammatory musculoskeletal complaints.

## Introduction

Systemic lupus erythematosus (SLE) is a chronic autoimmune inflammatory disease characterized by multisystem involvement and production of autoantibodies [1]. Its incidence is significantly higher in women, with a female-to-male ratio of approximately 9:1 [1,2]. Male patients often present with more severe or atypical clinical features, contributing to diagnostic challenges [3]. Musculoskeletal manifestations occur in up to 90% of patients [4,5], but isolated inflammatory knee pain as an initial presentation is unusual. Advances in imaging modalities such as MRI and PET-CT have improved early detection of inflammatory activity in autoimmune diseases [6-9].

This case illustrates the diagnostic complexity of SLE in a young male presenting primarily with inflammatory knee pain.

## Case presentation

**Figure 1:**
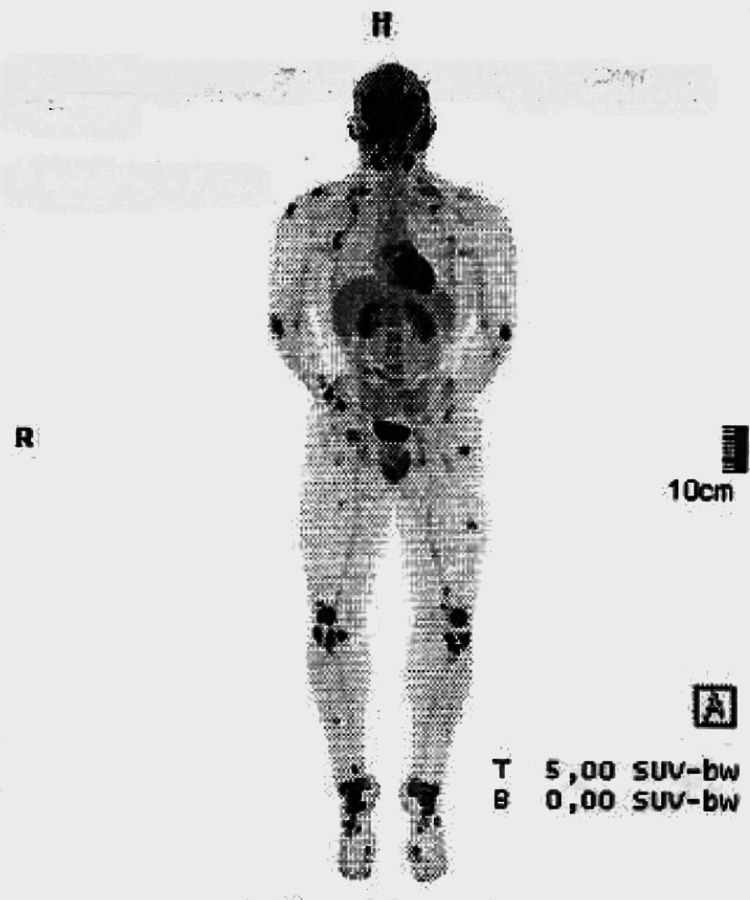
¹⁸F-fluorodeoxyglucose positron emission tomography-computed tomography (¹⁸F-FDG PET-CT) demonstrating multiple symmetric hypermetabolic osteo-medullary foci

A 19-year-old male with a history of epilepsy and a maternal history of SLE presented with bilateral inflammatory knee pain lasting three months. The pain improved with activity and worsened at rest. MRI demonstrated patellofemoral chondromalacia and multiple popliteal ganglion-like formations. Given the unusual MRI findings and the inconclusive clinical presentation, an ¹⁸F-fluorodeoxyglucose positron emission tomography-computed tomography (¹⁸F-FDG PET-CT) was performed, which revealed symmetric hypermetabolic osteo-medullary foci and multiple hypermetabolic subcutaneous lesions (Figure 1), raising suspicion of a systemic inflammatory disease.

**Figure 2:**
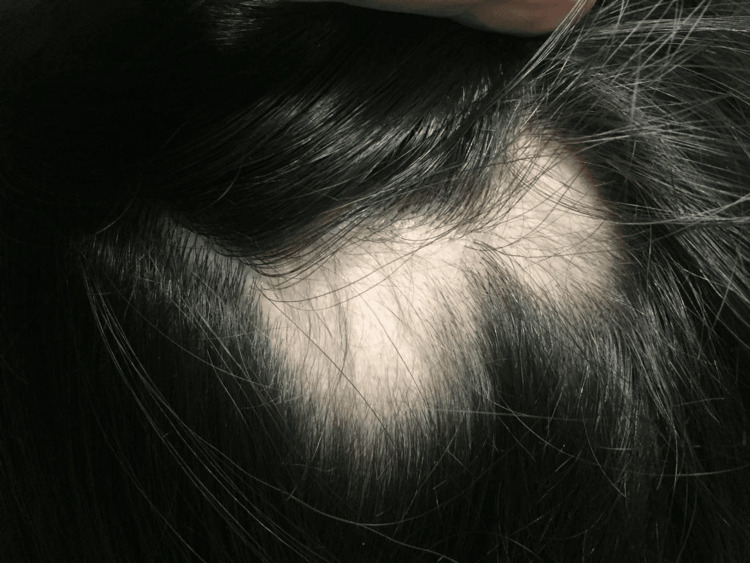
Non-scarring alopecic patches with preserved follicular ostia

**Figure 3:**
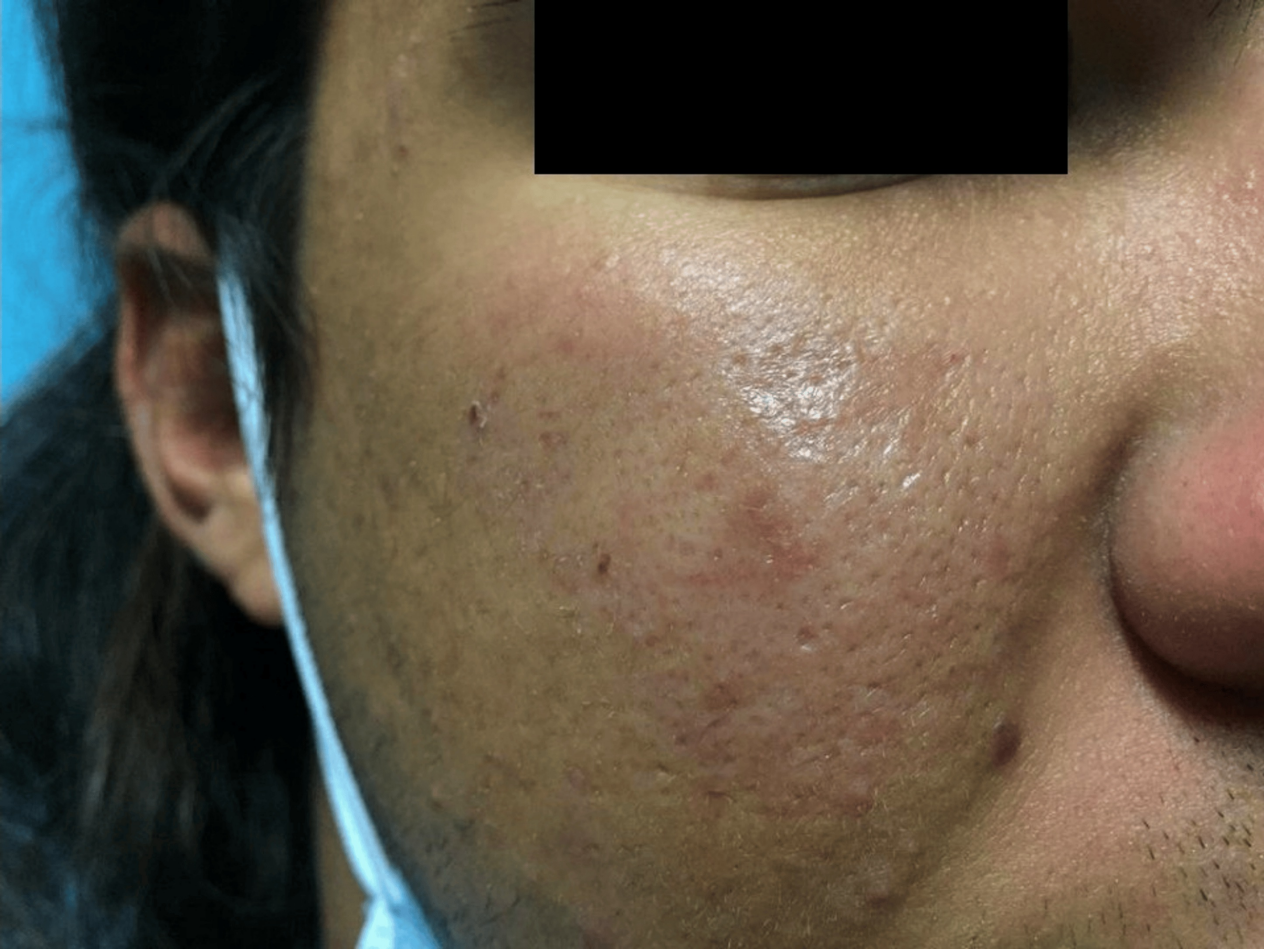
Indurated subcutaneous plaque over the right malar region

On examination, the patient demonstrated multiple non-scarring alopecic patches on the scalp (Figure 2) and a subcutaneous plaque over the right malar region (Figure 3). There was no evidence of synovitis, joint effusion, or deformity. Laboratory evaluation revealed persistent leukopenia (<3,800/µL) since the age of 16 years, predominantly due to neutropenia, along with hypocomplementemia (low C3 and C4) and positive antinuclear antibodies at a titer of 1:160 with a speckled pattern. Anti-double-stranded DNA, anti-Smith, anti-RNP, and anti-Ro/La antibodies were negative, and inflammatory markers were within normal limits. Lupus anticoagulant testing was also negative.

MRI of the face demonstrated thickening of the superficial malar tissues and a poorly defined short tau inversion recovery (STIR) hyperintense lesion (48 × 25 mm) with peripheral enhancement. Biopsy of the malar lesion revealed fibro-adipose tissue with osteonecrosis, myxoid degeneration, and a nonspecific inflammatory infiltrate. Importantly, the histopathological findings were not diagnostic, and the diagnosis of systemic lupus erythematosus was established based on the overall clinical, laboratory, and imaging features rather than biopsy results, which is a recognized limitation in SLE.

**Figure 4:**
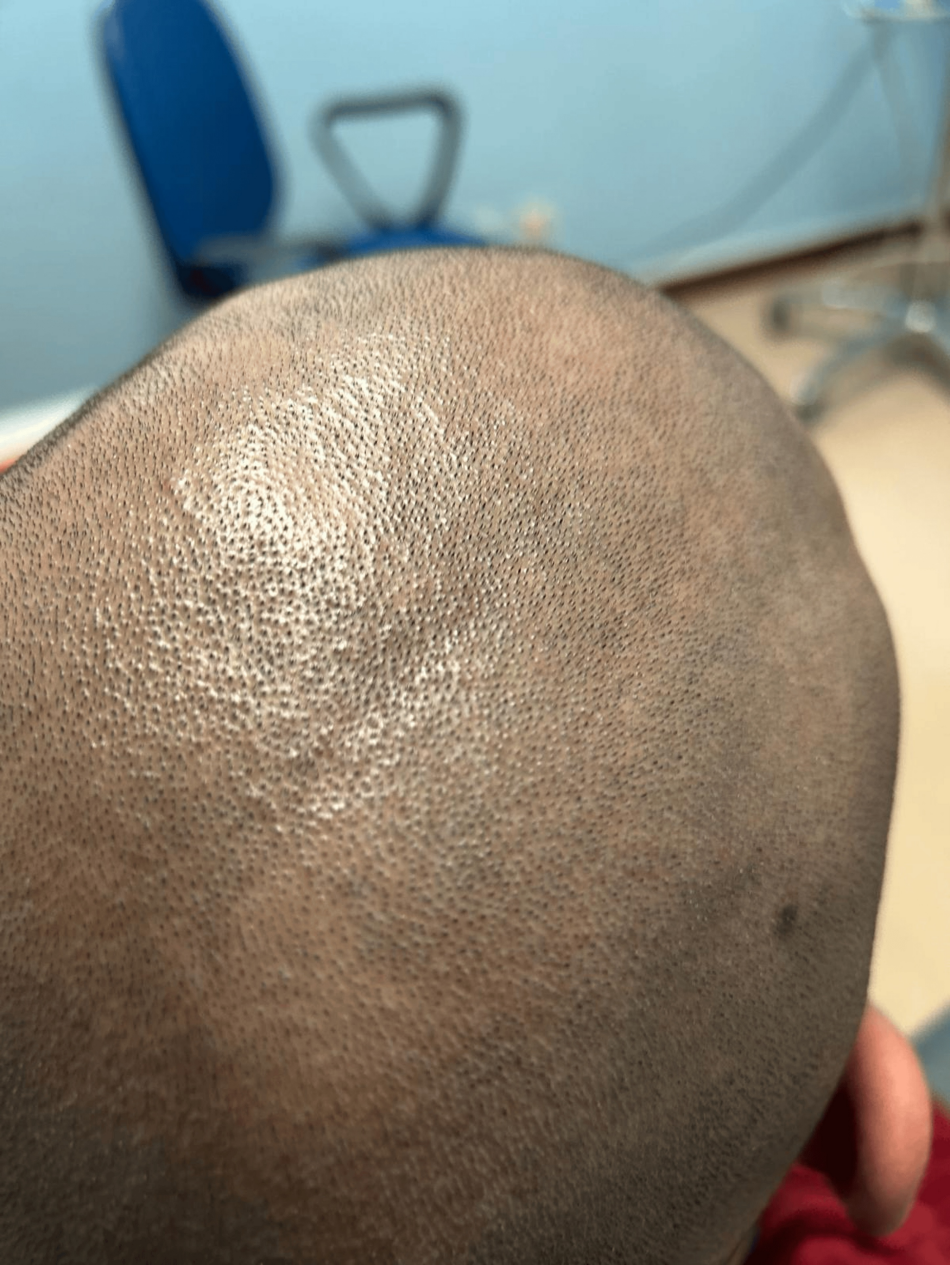
Significant regrowth of scalp hair after 12 months follow-up

Applying the 2019 European Alliance of Associations for Rheumatology (EULAR)/American College of Rheumatology (ACR) classification criteria resulted in a score of 14 points, confirming the diagnosis of SLE [1]. Based on disease activity assessment, the patient had an estimated Cutaneous Lupus Erythematosus Disease Area and Severity Index (CLASI) activity score of 4-5, driven predominantly by non-scarring alopecia, with no evidence of cutaneous damage, and a British Isles Lupus Assessment Group (BILAG)-2004 profile showing moderate mucocutaneous and hematologic involvement (grade B) with mild musculoskeletal disease (grade C). In light of this activity profile, treatment with hydroxychloroquine 200 mg/day was initiated after ophthalmologic evaluation. At 12-month follow-up, the patient reported improvement in knee pain, stabilization of cutaneous lesions and a significant regrowth of scalp hair (Figure 4).

## Discussion

Musculoskeletal involvement is the most frequent presenting manifestation of SLE, occurring in up to 90% of patients [4,8]. However, initial presentation with isolated bilateral inflammatory knee pain in a young male is uncommon and may contribute to diagnostic delay. Although SLE is less prevalent in males, affected patients often exhibit more atypical or severe disease phenotypes, further complicating early recognition [3].

In this case, ¹⁸F-FDG PET-CT demonstrated symmetric osteomedullary hypermetabolic activity and subcutaneous uptake (Figure 1), supporting a systemic inflammatory process and aligning with previously reported findings in SLE [5,6]. PET-CT is not a standard diagnostic tool for SLE, as FDG uptake is nonspecific, associated with significant cost, and involves radiation exposure. Nevertheless, in selected atypical presentations, PET-CT may be instrumental in uncovering occult systemic inflammation and prompting further targeted evaluation [9].

Given the imaging findings, several alternative diagnoses were considered, including chronic recurrent multifocal osteomyelitis, sarcoidosis and hematologic malignancy. Chronic recurrent multifocal osteomyelitis was deemed unlikely due to the absence of relapsing bone pain since childhood and lack of characteristic imaging progression. Sarcoidosis was considered but excluded based on the absence of pulmonary, lymph node, or granulomatous involvement. Hematologic malignancy was unlikely given the symmetric uptake pattern, absence of constitutional symptoms, and stable laboratory findings. The combination of cutaneous manifestations, hematologic abnormalities, hypocomplementemia, positive antinuclear antibodies, and fulfillment of EULAR/ACR classification criteria ultimately supported the diagnosis of SLE over these alternatives.

Cutaneous findings, including non-scarring alopecia (Figure 2) and subcutaneous plaques consistent with lupus panniculitis (Figure 3), are recognized manifestations of SLE and contribute to disease classification [1]. The osteomedullary and subcutaneous FDG uptake observed on PET-CT may reflect cytokine-mediated immune activation, immune-complex deposition, and inflammatory infiltration of bone marrow and adipose tissue, mechanisms that are increasingly recognized in lupus pathophysiology. Histopathologic evaluation of the malar lesion was nonspecific, underscoring that tissue biopsy in SLE may lack diagnostic specificity and that diagnosis frequently relies on integration of clinical, laboratory, and imaging findings rather than histology alone.

Based on objective disease activity assessment using CLASI and BILAG-2004 indices, antimalarial therapy was initiated. After 12 months of hydroxychloroquine treatment, the patient demonstrated sustained improvement in musculoskeletal symptoms and near-complete resolution of lupus-associated non-scarring alopecia (Figure 4), reflecting a favorable and durable therapeutic response.

This case highlights the importance of systematically excluding alternative causes of osteoarticular pain in young patients with inflammatory symptoms, particularly when imaging reveals systemic abnormalities. In such atypical presentations, careful integration of clinical features, laboratory findings, and selected imaging modalities is essential to avoid delayed diagnosis of underlying autoimmune disease.

## Conclusions

Systemic lupus erythematosus can present atypically in young male patients. Persistent inflammatory knee pain, associated cutaneous manifestations, and systemic imaging abnormalities such as those observed on PET-CT should prompt consideration of autoimmune disease after exclusion of alternative etiologies. Early diagnosis and timely initiation of hydroxychloroquine may improve clinical outcomes.
